# Preventive Dentistry

**Published:** 1919-11

**Authors:** Russell W. Bunting

**Affiliations:** Ann Arbor, Mich.


### PREVENTIVE DENTISTRY

BY RUSSELL W. BUNTING, D.D.Sc., ANN ARBOR, MICH.

Dentistry today is on trial. Accusation has been brought against her that she is a menace to public health, that many of the things which she has done in the past to save teeth, have resulted in disease and disaster to the individual, and that beautiful restorations and extensive reconstructions which she has made are in realty "whited sepulchres" from which insidious poisons continually seep and spread throughout the body. She has, in fact, been challenged to show the integrity of her purpose and proof of the safety of various dental procedures. What is safe and sane in dentistry and when does it cease to be a menace?

Dentistry has responded to the challenge and is seeking to meet the issue. She recognizes that systemic infections may gain entrance to the body through the oral tissues and that certain of these are connected with dental operative procedures; also that danger lies in many peridental infections which have formerly been overlooked or given scant consideration. So forcibly have these facts been brought home to her that she can no longer evade them. It has therefore become necessary that she inquire very closely into the relation of systemic infection to oral health, and to recognize her whole system of operative dentistry to meet the new situation. It is very evident that the great majority of infections which enter the body through the oral tissues do so through two specific channels: one, periapical infections about the roots of devitalized teeth, and the other, pyorrhetic affections of the peridental tissues. It is toward these two types of oral infection that attention has been directed.

In the effort to eradicate the first of these infections (periapical), intensive study has been made of the treatment of pulpless teeth. Strenuous efforts have been made to perfect the technic of pulp canal operations in the attempt to devise a method which is above reproach. In spite of all that has been done in this regard, the attempt has not resulted so far in unqualified success. A few operators, by infinite pains and most careful technic, have attained a high degree of success; but the great majority of dentists are still treating and filling root-canals in a manner that is open to serious criticism. And, indeed, it will never be otherwise until an easy, certain, foolproof method is devised by which the average man, as well as the expert, shall be able to treat and fill root-canals in a manner that is above reproach. Then, and not until then, may dentistry justify the retention of all devitalized teeth.

A relatively small number of practitioners have developed the requisite skill to treat pyorrhetic affections, so that the progress of that disease is permanently arrested and the peridental infections are eradicated. But the great majority of dentists are treating these conditions very indifferently; either they neglect them entirely or, if they attempt to treat them at all, they do not carry them through to a successful completion. Dentistry can never justify the retention of pyorrhetic teeth unless she adopts measures which will make them unquestionably safe as regards infection.

There are then for dentistry two courses of action if we would avoid criticism and condemnation: one, the perfection of a technic in the treatment of pulpless teeth and of pyorrhetic affections so that we can say with authority that the possibility of systemic infection has been removed; the other, extraction of all diseased or suspected teeth. This drastic ultimatum has been delivered to the dental profession by our medical confreres, by our patients, and by the public at large, and no matter how severe it may seem, or how difficult the execution may be the situation nevertheless, cost what it may, must be met.

There is, however, a third course of action which is much more reasonable and *efficient* than the other two and which, if universally adopted, would most *effectually* meet the situation. Could we keep the pulps of all teeth alive and could we keep the peridental tissues in health, we would strike a blow at the root of the difficulty and would prevent the inception of these peridental infections which we have learned to fear. By such measures we would obtain an unquestionable guarantee of the safety of dental procedures and would establish an irrefutable alibi for the mouth as a menace to general health. We may well consider at this time any measures for prevention which have proved to be of value.

When we consider the preservation of dental pulps, it must be admitted that by far the greatest cause of the death of that tissue is dental caries. Pulp conservation, then, may best be accomplished by the limitation of caries inception and the filling of cavities which appear in the teeth before they become of sufficient size to involve the pulp. Volumes have been written upon the cause, course and prevention of dental caries but in all that has been contributed to the subject, there is but one practical method suggested for its prevention which has proved to be of value, namely, oral hygiene in its broadest sense. Dental caries has been recognized as destruction of the tooth by acids resulting from the fermentation of carbohydrates; therefore it is reasonable to expect that the continuous cleansing of the mouth of fermentable food stuffs and the reduction of infections will tend to decrease the inception of caries. By practical demonstration this has proved to be true. Perhaps in the future better and more definite methods of caries control will be determined, but at the present time the practice of oral prophylaxis and oral hygiene, as applied to every department of operative dentistry, comprises the most efficient measures of caries prevention.

These measures consist in more than mere cleansing of the teeth. This is but the first step in the process and should be followed by a thorough polishing of the teeth so that they will offer less natural retention for food debris and bacterial plaques. All fillings, crowns, bridges, and so forth, should be so shaped and polished that they will offer the least possible retention to foodstuffs, and in every way the attempt should be made to make the mouth as nearly self-cleansing as possible. The patient must be instructed as to personal care of the mouth, and by encouragement and moral suasion, a cordial cooperation in this work must be gained. The suggestion of certain diets is often of value, such as the selection of hard and tough foods, the avoidance of soft, pappy and surgary substances, and the eating of acid foods or fruits at the end of every meal. Unless all of these measures of oral hygiene in its broadest sense are carried out, the simple cleansing of the teeth will prove of little avail.

It is with the children that the greatest measure of success may be attained. The mouths of the adults are already sadly mutilated as the result of past neglect and are encumbered with various forms of restorations, many of which are of indifferent character and questionable safety. But the children present a field of endeavor which is fresh and as yet unspoiled by the ravages of dental disease. If their mouths are kept clean, they will have far less caries than their parents had before them, and if they are continually watched, the cavities which do occur may be discovered and successfully filled before they have involved the pulps. Thus it is possible and practicable to bring the youth to manhood and womanhood with a full normal complement of teeth in which every pulp is alive. For them the problem of keeping their mouths in health and free from insidious infections will be far simpler than that of the present generation. Perhaps this form of practice is best accomplished by those of our profession who are confining their attention to the treatment of children. But splendid as this work may be, the number of children who may have such attention is extremely small compared with the masses who receive little or no dental care. Service of this kind is too important to be limited to the wealthy classes, but must be developed rather as a public health measure and placed within reach of all. To accomplish this, the public schools offer the most strategic avenue by which all children may be reached, and it is through them that the program of universal dental health must be carried out. Already rapid strides have been taken in this direction and everywhere attempts are being made to institute school dental clinic and dental examinations for all school children. But the field is large and the surface has only been scratched, while the supply of competent workers is pitifully small and inadequate. Additional impetus has been given this work recently by the advent of a corps of young women trained to serve in the field of preventive dentistry—the dental hygienists.

The work of these women in the public schools of other states has fully demonstrated their value, and by an enactment of our legislature we trust that they may become a reality in this state. Time and effort alone can show what may be accomplished by their assistance in this colossal task. This we know: that preventive dentistry for children is fast becoming a large and important branch of dental service and of public health as well; that it is practicable, beneficial and entirely defensible, and that it is our duty as a profession to support it and advance it in every way possible until it has reached every child in every school in the land.

Preventive dentistry for adults is somewhat more difficult of accomplishment, especially when the mouth has already been severely attacked by dental disease and contains extensive restorations. But in the cases which present themselves in the daily routine of dental practice very beneficial results may be obtained when all operative procedures are made to conform to the principles of oral hygiene. A general reorganization of the mouth to a condition in which it is possible for the patient to keep it clean and teaching him how to accomplish it will always result in a reduction of oral infections and a decrease and often an entire prevention of the inception of dental caries. Periodic examinations are also necessary for the adult as well as children in order that assistance may be given in keeping the mouth clean and that cavities may be detected and filled while small for the sake of pulp conservation.

The second class of oral infections, the peridental affections, are much more easily and definitely controlled. The various disease processes known as pyorrhea consist essentially in a breaking down of the hard and soft tissues about the tooth by infective organisms. The inception of the process is not so much due to the presence of a specific type of organism as it is to a lowering of the resistance of peridental tissues to such infective agents. When in health, the oral mucous membranes form a resistant barrier to keep infections from gaining entrance to the underlying tissues. The most vulnerable spots in this protecting wall are the gingival areas where the mucous membranes surround each tooth. Here nature has intended that the soft and hard tissues shall be so closely connected that infection can not break through, and as long as the gingival tissues remain in a healthy state, this is true. But by reason of various local irritants, such as calculus, constant infection, foodstuffs, overhanging fillings, and so forth, the gingival circulation becomes disturbed; active and passive hyperemia, stasis and inflammation, follow in rapid succession, and the normal tone and resistance of these tissues are lost. The various forms of gingivitis and pyorrhetic affections which follow, effectually open the way by which oral infections may penetrate the deeper tissues and be carried throughout the system. It is true that occasionally these conditions arise from a lowering of resistance by virtue of some systemic fault, but the far greater majority owe their beginning to local irritations. Could we but keep these gingival tissues free from irritations, they would almost invariably remain in health. So that although the treatment of advanced pyorrhea is a difficult and highly specialized procedure, the prevention of pyorrhetic affections may be most easily and surely accomplished. In the early stages certain definite signs appear in the gingival tissues, such as changes in color, in form, or in density, which are so apparent that even "he who runs may read." And when these deviations from normal are noted, if the operator turns his attention to removing the local irritations, which are disturbing these tissues, a rapid return to normal tone and resistance may easily be obtained. Indeed, these measures are so closely related to the principles of oral hygiene which we have just stated that if they be continued to include the necks of the teeth beneath the free margin of the gum, they will constitute an efficient method of preventing both classes of dental disease.

Oral hygiene has come to occupy a position of importance in the field of dentistry. This department of dental practice which has been given but minor consideration and assiduously avoided by the majority of practitioners in the past—this stone which was rejected by the builders of our profession in earlier days, bids fair to become the corner stone of the new structure which dentistry will build in the future. No longer may oral hygiene be considered as the mere cleaning of teeth, but by virtue of its manifest importance, it dominates all operative procedures and demands that they restore the dental organs to their full normal function to the end that the mouth may offer the least possible retention to foodstuffs and infectious materials. In return, oral hygiene offers to dentistry a most reasonable and defensible alibi to the charges which are now being made against her.

As in a recent editorial in the *Dental Cosmos*, Dr. Kirk, in discussing the question of preventive dentistry made the following statement: "The hope of the future in dentistry, as well as medicine, is prevention—and the hope of prevention of dental diseases to any considerable degree in the future lies in oral prophylaxis systematically and persistently prosecuted."—*Journal N. D. A.*

